# Anecdote of the Pay of Physicians

**Published:** 1846-12

**Authors:** 


*Anecdote of the Pay of Physicians.*—One day, old Kien-Long, Emperor of China, asked George Stanton how Doctors were paid in England. When he comprehended the system, he exclaimed, "can there be a single Englishman in good health? Let me tell you how I treat my physicians. There are four to whom my health is confided. They receive a certain sum every week; but the moment I am taken sick, their salaries are stopped until I get better. It is scarcely necessary for me to add that my complaints do not last long."—*S. Med. & Surg. Jour.*

